# Regulatory potential of post-translational modifications in bacteria

**DOI:** 10.3389/fmicb.2015.00500

**Published:** 2015-05-28

**Authors:** Christophe Grangeasse, Jörg Stülke, Ivan Mijakovic

**Affiliations:** ^1^Bases Moléculaires et Structurales des Systèmes Infectieux, UMR 5086, Centre National de la Recherche Scientifique, University of LyonLyon, France; ^2^Department of General Microbiology, Institute for Microbiology and Genetics, University of GöttingenGöttingen, Germany; ^3^Systems and Synthetic Biology Division, Department of Biology and Biological Engineering, Chalmers University of TechnologyGöteborg, Sweden

**Keywords:** phosphorylation, S-thiolation, dehydration, hydroxylation, N-glycosylation, protein kinases, antimicrobial peptides, proteomics

Bacteria are often viewed as simple organisms, with very basic and robust cellular regulation, optimized for rapid growth. While they certainly fit that description, bacteria also possess an amazing capacity for adaptation, and diversity of survival strategies, including variations of cell morphology, size, mode of growth and developmental behavior. Post-translational modifications (PTMs) of proteins contribute significantly to bacterial adaptability and cell cycle control. Research on PTMs in bacteria started with the assumption that they lack many features regularly found in more complex organisms. However, ongoing investigation keeps revealing new types of PTMs of bacterial proteins. This research topic gathers a number of articles discussing the advanced methods for systematic study of bacterial PTMs, approaches to utilize PTMs for biotechnological purposes, and revealing new cellular functions controlled by PTMs.

Most bacterial PTMs are dynamic and reversible. This allows the cell to exploit them as regulatory devices. It also means that the full understanding of the cellular roles of different PTMs necessitates global, quantitative and time-resolved studies. One seminal paper in this topic reports a novel proteomics approach for such quantitative studies: the Intensity Based Absolute Quantitation (iBAQ). Using this approach, the authors have quantified the expression and the occupancy of various PTM sites in the proteome of *Escherichia coli* (Soufi et al., 2015). The authors report remarkable differences in expression and occupancy of PTMs sites under different growth conditions. The dataset comprised 2300 proteins, which is close to 90% of the expressed proteome. This is an important landmark, since proteomics in general has not yet attained the same level of coverage that global transcriptome studies can achieve.

Among different bacterial PTMs, protein phosphorylation is the most extensively studied one, and in this topic it features prominently. Several papers present global studies of protein phosphorylation in bacteria (Spät et al., 2015), including important bacterial pathogens (Fortuin et al., 2015; Nakedi et al., 2015). The focus on pathogens is understandable, since protein phosphorylation, and several other PTMs, are heavily involved in different infection strategies displayed by bacterial pathogens (Michard and Doublet, 2015). In addition to phosphoproteome studies, interactomics is also featured as a useful approach to chart the phosphorylation-based regulatory networks. It enables researchers to trace the connections among protein kinases, phosphatases, and their substrates (Shi et al., 2014a). This approach for reconstructing phosphorylation networks highlights the capacity of bacterial proteins kinases from different families to interact with, and phosphorylate, each other (Shi et al., 2014b). Protein-tyrosine kinases, and their various roles in bacterial physiology were in the focus of several review and opinion articles in this topic (Barák, 2014; Gerwig and Stülke, 2014; Mijakovic and Deutscher, 2015).

Other topic contributions highlight the broad spectrum of PTMs involved in key cellular processes. In particular, novel modifications are discussed: redox regulation via reversible S-thiolation (Loi et al., 2015), post-translational hydroxylation (van Staalduinen and Jia, 2015) and the role of citrullination for the interaction between bacteria and human mucosal surfaces (Sofat et al., 2015). Several experimental papers report studies on post-translationally modified antimicrobial peptides known as lantibiotics. These are ribosomally synthesized peptides which can efficiently inhibit the growth of Gram-positive bacteria. A study by Zhou et al. (2015) described an engineering strategy to make the lantibiotics more effective in inhibiting several important bacterial pathogens. In another study, Khusainov et al. (2015) describe the active site of the nisin dehydratase. This enzyme, essential for the production of the lantibiotic nisin, converts serines, and threonines, to dehydroalanine and dehydrobutyrine residues, respectively. Interestingly, bacterial PTM systems, such as the N-glycosylation machinery, are also being exploited and engineered to facilitate the production of recombinant vaccines (Garcia-Quintanilla et al., 2014). Another focal point of the topic are the PTMs targeting the PII proteins, which are the key signal transduction proteins involved in the control of nitrogen metabolism in bacteria and archaea (Radchenko et al., 2014; Merrick, 2015). Finally, Stannek et al. (2015) contributed a study on a regulatory mechanism involving arginine phosphorylation and regulated proteolysis.

In conclusion, the contributions in this topic reflect the diversity of bacterial PTMs. Several studies highlight one important emergent feature of PTM systems: their capacity to interact with each other, creating an additional level of complexity in the cellular regulation. This is one of the key features of bacterial PTM systems and a challenge which future studies will have to address.

## Conflict of interest statement

The authors declare that the research was conducted in the absence of any commercial or financial relationships that could be construed as a potential conflict of interest.
